# Diffuse Decreased Gray Matter in Patients with Idiopathic Craniocervical Dystonia: A Voxel-Based Morphometry Study

**DOI:** 10.3389/fneur.2014.00283

**Published:** 2015-01-08

**Authors:** Camila C. Piccinin, Luiza G. Piovesana, Maria C. A. Santos, Rachel P. Guimarães, Brunno M. De Campos, Thiago J. R. Rezende, Lidiane S. Campos, Fabio R. Torres, Augusto C. Amato-Filho, Marcondes C. França, Iscia Lopes-Cendes, Fernando Cendes, Anelyssa D’Abreu

**Affiliations:** ^1^Neuroimaging Laboratory, University of Campinas, Campinas, Brazil; ^2^Department of Neurology, University of Campinas, Campinas, Brazil; ^3^Department of Medical Genetics, University of Campinas, Campinas, Brazil; ^4^Department of Radiology, University of Campinas, Campinas, Brazil

**Keywords:** craniocervical dystonia, voxel-based morphometry, gray matter, cervical dystonia, segmental dystonia, neuroimaging

## Abstract

**Background:** Recent studies have addressed the role of structures other than the basal ganglia in the pathophysiology of craniocervical dystonia (CCD). Neuroimaging studies have attempted to identify structural abnormalities in CCD but a clear pattern of alteration has not been established. We performed whole-brain evaluation using voxel-based morphometry (VBM) to identify patterns of gray matter (GM) changes in CCD.

**Methods:** We compared 27 patients with CCD matched in age and gender to 54 healthy controls. VBM was used to compare GM volumes. We created a two-sample *t*-test corrected for subjects’ age, and we tested with a level of significance of *p* < 0.001 and false discovery rate (FDR) correction (*p* < 0.05).

**Results:** Voxel-based morphometry demonstrated significant reductions of GM using *p* < 0.001 in the cerebellar vermis IV/V, bilaterally in the superior frontal gyrus, precuneus, anterior cingulate and paracingulate, insular cortex, lingual gyrus, and calcarine fissure; in the left hemisphere in the supplementary motor area, inferior frontal gyrus, inferior parietal gyrus, temporal pole, supramarginal gyrus, rolandic operculum, hippocampus, middle occipital gyrus, cerebellar lobules IV/V, superior, and middle temporal gyri; in the right hemisphere, the middle cingulate and precentral gyrus. Our study did not report any significant result using the FDR correction. We also detected correlations between GM volume and age, disease duration, duration of botulinum toxin treatment, and the Marsden–Fahn dystonia scale scores.

**Conclusion:** We detected large clusters of GM changes chiefly in structures primarily involved in sensorimotor integration, motor planning, visuospatial function, and emotional processing.

## Introduction

Dystonia is a movement disorder characterized by involuntary sustained or intermittent muscle contractions causing abnormal, often repetitive, postures or movements (1). Blepharospasm (BSP), oromandibular, lingual, laryngeal, and cervical dystonia (CD) are common forms of adult-onset dystonia. The term craniocervical dystonia (CCD) includes each of these conditions as well as their combinations. Although subjects with different manifestations of CCD probably have underlying differences in brain pathology, clinically there is a significant overlap. Several authors have studied CCD as a group suggesting the presence of common pathological mechanisms (2–7).

Classically considered, a manifestation of basal ganglia dysfunction, neuroimaging techniques have demonstrated structural, functional, and molecular brain abnormalities in areas such as the sensorimotor cortex and cerebellum (2, 7).

Voxel-based morphometry (VBM) allows for the evaluation of whole-brain structural changes by comparing two or more groups of MRI images (8). There have been eight published studies on morphometric gray matter (GM) abnormalities in patients with CD or BSP using VBM (9–16). The methodologies used were heterogeneous, and the results were variable without a consistent pattern of structural changes (Table 1). Moreover, we recently performed a cerebellar analysis in CCD using a specific template atlas for cerebellum, which found GM increase in the anterior cerebellar lobe and GM decrease in the posterior cerebellar lobe. Despite these interesting findings, in this technique, the infratentorial structures are isolated from the surrounding tissue (to avoid being biased by them), and therefore, the supratentorial areas were not studied (7). Thus, on the basis of previous conflicting results in the literature, our aim was to investigate morphological abnormalities in GM in CCD in order to better elucidate and characterize them using the most recent version of the software for VBM analysis as well as a larger sample.

**Table 1:** **Comparison of methodologies and results of previous VBM studies in craniocervical dystonias**.

Ref.	MRI	SPM	FWHM	Mod	Dys	PTS	HC	*p* value	Decreased GM	Increased GM
Draganski et al. (9)	1.5 T	SPM99	10 mm	No	CD	10	10	< 0.05 correct	R caudal SMA, R visual cortex, R dlPFC	R GPi, bilateral motor cortex, cerebellar flocculus
Etgen et al. (13)	1.5 T	SPM2	12 mm	No	BSP	16	16	< 0.001 uncorrected	L inferior parietal lobule	Bilateral putamen
Egger et al. (10)	1.5 T	SPM2	12 mm	No	CD	11	11	< 0.05 correct		Bilateral orbitofrontal cortex, R GPi, medial frontal gyrus, L SMA, and cingulate gyrus
Obermann et al. (12)	1.5 T	SPM2	12 mm		BSP	11	11	< 0.05 correct	Bilateral thalamus, putamen	Bilateral caudate head, cerebellum
					CD	9	9	< 0.05 correct	Bilateral putamen, superior temporal lobule	Bilateral caudate head, thalamus, L posterior cerebellar lobe, and superior temporal lobule
Suzuki et al. (14)	1.5 T	SPM8	9 mm	No	BSP	32	48	< 0.05 correct		Bilateral sensory-motor cortices, L cingulate
Martino et al. (15)	3 T	SPM8	8 mm	Yes	BSP	25	24	< 0.05 correct	L superior temporal gyrus, postcentral gyrus	R middle frontal gyrus
								< 0.001 uncorrected		Bilateral superior frontal gyrus, R middle frontal gyrus, L anterior cingulate
Pantano et al. (16)	1.5 T	SPM5	12 mm	Yes	CD	19	28	< 0.05 correct	Bilateral premotor and primary sensory-motor cortices, L caudate head, putamen	
Prell et al. (11)	1.5 T	SPM2	8 mm	Yes	CD	24	24	< 0.001 uncorrected	L precentral, SMA, medial temporal gyrus, and R somatosensory association cortex	L GPi, frontal eye field, R claustrum, putamen, and bilateral medial surface of occipital lobe

*FWHM, full width half maximum; Mod, modulation; Dys, type of dystonia; PTS, number of patients; dlPFC, dorsolateral prefrontal cortex; GPi, globus pallidus internus*.

## Materials and Methods

### Subjects

The Institutional Review Board of our University Hospital approved the study, and all subjects signed an informed consent prior to participation.

We included 27 subjects (mean age of 54.18 ± 11.70 years) with a clinical diagnosis of idiopathic CCD recruited at the Movement Disorders Outpatient Clinic, the Dystonia Outpatient Clinic, and the Neurogenetic Service of the University of Campinas (UNICAMP) University Hospital. All patients included were negative for DYT-1 and DYT-6 mutations. We performed a detailed clinical evaluation, which included a review of the medical history, duration of botulinum toxin treatment (BoNT), physical and neurological examination, and the Marsden–Fahn Scale (MFS). When conducting the MRI exam, all patients were at the peak of action of botulinum toxin, so that the images were obtained with a lower chance of motion artifacts. All clinical data are detailed in Table 2. The clinical presentation of dystonia is described in Table 3.

**Table 2:** **Clinical data of patients and control subjects**.

	Patients	Controls
Gender (male)	9	18
(female)	18	36
Age (years)	54.18 ± 11.70 (29–75)	54.00 ± 11.47 (29–77)
Disease duration (years)	11.37 ± 6.78 (2–27)	–
BoNT (years)	4.20 ± 3.82 (0–12)	–
MSF score	5.13 ± 2.61 (2.5–10.5)	–

*BoNT, botulinum toxin treatment duration; MSF score, score in Marsden–Fahn scale*.

**Table 3:** **Clinical presentation of CCD in study participants**.

Distribution	Localization	*N* patients	%
Focal	Cervical (C)	13	48.1
	Blepharospasm (B)	2	7.4
	Oromandibular (O)	2	7.4
Segmental	B + O	3	11.1
	B + O + C	5	18.5
	O + C	1	3.7
	O + laryngeal	1	3.7
Total	Total	27	100%

We included 54 healthy controls, with no history of neurological disorders, no family history of dystonia, and a normal neurological examination (mean age of 53.92 ± 11.47 years). Each patient was matched with two control subjects of the same gender and age of at most 2 years apart.

### MRI acquisition

Images were acquired at a 3 T Achieva MR unit-PHILIPS Intera^®^, release 2.6.1.0. In addition to the usual diagnostic sequences, we obtained volumetric T1-weighted image, with isotropic voxels of 1 mm^3^, acquired in the sagittal plane (1 mm thick, flip angle, 8°, TR 7.1, TE 3.2, matrix 240 × 240, and FOV 240 × 240 mm).

### Image pre-processing

Prior to any imaging processing, an experienced neuroradiologist evaluated all images in a blinded fashion for controls and patients, to assure image quality and the absence of significant brain pathology or artifacts. Then, we aligned all the images according to the anterior commissure and rotated those images in the sagittal, axial, and coronal planes according to the same references, when necessary, in order to provide the most homogeneous sample possible.

### Voxel-based morphometry analysis

The VBM processing entailed four main steps: spatial normalization of all images to the same stereotactic space, segmentation into GM, white matter, and cerebrospinal fluid, re-slicing into an atlas space (modulation), and smoothing. The difference between our previous and present study is that in the first one our aim was to investigate morphological changes of the cerebellum, since functional studies had reported abnormalities in dystonic patients. For this purpose, we used the spatially unbiased infratentorial template (SUIT tool), which isolates the infratentorial structure from the surrounding tissue before the spatial normalization of VBM. The idea was to prevent the supratentorial structures from biasing the results. Moreover, the normalization with the SUIT template avoids the stretching of the cerebellum in *z*-direction and leads to a better overlap between the cerebellar lobules. Therefore, the present study aims to complement the previous one, using a whole-brain template since the supratentorial areas are also involved in the pathophysiology of CCD.

In the present study, the images were automatically normalized, segmented, and modulated by SPM8 (Statistical Parametric Mapping) from MATLAB R2011b platform, according to technical and methodological protocols (www.fil.ion.ucl.ac.uk/spm/). The modulation compensates for the effect of the non-linear spatial normalization by multiplying the voxel intensities for the local value in the deformation field from normalization. This procedure allows the use of a small filter in the smoothness and the measurement of GM volume instead of GM density only. We next performed a homogeneity test to check the standard deviation across the sample using the covariance of the images. We also re-sliced the images by interpolation of the voxels in order to generate a final voxel size of 1 mm^3^.Finally, the GM probability images were smoothed with 10 mm full width half maximum (FWHM) isotropic Gaussian kernel filter in SPM8/DARTEL to satisfy the Gaussian distribution assumption for statistical analysis of regional differences. This process minimizes effects due to residual differences in functional and gyral anatomy during intersubject averaging and renders the data more normally distributed. As our analysis is based on unbalanced sample sizes, a filter with a greater extent is needed to avoid a greater number of false positives. Considering that our subjects are matched and *p* < 0.001, we used a FWHM of 10 mm instead of the 12 mm filter, due to the increased risk of false negative results (17). Using SPM8/DARTEL, a two-sample *t*-test corrected for subjects’ age was created for voxel-by-voxel analysis and detection of GM differences between the groups. In the contrast manager, we determined one contrast for GM atrophy and one for GM excess. Two different statistical parametric maps were generated: one corrected for false discovery rate (FDR), *p* < 0.05 and other for *p* < 0.001 uncorrected. Automated anatomical labeling (AAL) was used for anatomical localizations.

We also attempted to correlate the GM volume of the patients with the following clinical data: age, disease duration, duration of BoNT, and the MFS score. This analysis was performed in SPM8 using separate regression analyses. In the contrast manager, we could establish positive and negative correlations.

## Results

**Figure 1:**
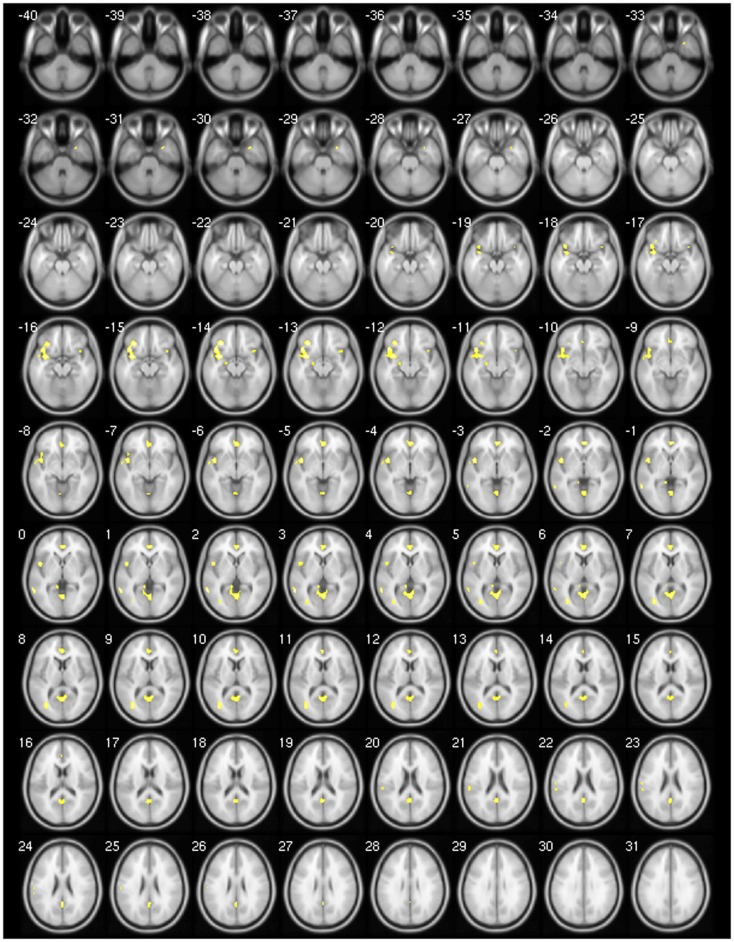
**Areas showing decreased GM in axial slices using. *p* < 0.001**.

We found decreased GM using *p* < 0.001 and clusters > 50 voxels in the cerebellar vermis IV/V, bilaterally in the superior frontal gyrus, precuneus, anterior cingulate and paracingulate, insular cortex, lingual gyrus, and calcarine fissure; in the left hemisphere in the supplementary motor area (SMA), inferior frontal gyrus (IFG), inferior parietal gyrus, temporal pole, supramarginal gyrus, rolandic operculum, hippocampus, middle occipital gyrus, cerebellar lobules IV/V, superior, and middle temporal gyri; and finally, in the right hemisphere, the middle cingulated and precentral gyrus (Table 4; Figure 1).

**Table 4:** **Areas of decreased GM using. *p* < 0.001**.

Gray matter (decrease) *p* < 0.001				
MNI peak co-ordinates		
*x*	*y*	*z*	Voxels	Areas (automated anatomical labeling)
2	40	−3	1151	Left anterior cingulate and paracingulate gyrus
			366	Right anterior cingulate and paracingulate gyrus
			97	Right superior frontal gyrus, orbital part
			88	Left superior frontal gyrus, orbital part
−34	4	−14	1006	Left temporal pole of superior temporal gyrus
			818	Left insula
			779	Left superior temporal gyrus
			351	Left inferior frontal gyrus, orbital part
			153	Left rolandic operculum
			97	Left inferior frontal gyrus, opercular part
0	−53	24	966	Left precuneus
			678	Cerebellar vermic lobule IV/V
			644	Left Calcarine fissure
			512	Left lingual gyrus
			275	Right lingual gyrus
			191	Right Calcarine fissure
			188	Left posterior cingulate
			160	Right precuneus
			59	Left cerebellar lobules IV/V
−34	−73	6	656	Left middle occipital gyrus
			74	Left middle temporal gyrus
−30	30	−14	419	Left inferior frontal gyrus, orbital part
−60	−51	2	397	Left middle temporal gyrus
38	16	−15	226	Right insula
−10	−41	2	156	Left lingual gyrus
			91	Left precuneus
−8	−1	64	145	Left supplementary motor area
−22	−9	−13	112	Left hippocampus
−58	31	21	108	Left supramarginal gyrus
			80	Left superior temporal gyrus
−40	−57	47	96	Left inferior parietal gyrus
14	−24	75	80	Right precentral gyrus
2	−17	49	65	Right middle cingulate

*Height threshold: *T* = 3.16; Extend threshold = 100 voxels; voxel size = 1 mm^3^; FWHM Gaussian filter = 10 mm*.

In order to refine our analysis, we performed the same analysis using FDR correction (*p* < 0.05), but we did not find any significant cluster of decreased or increased GM. In addition, there were no significant clusters of GM increased in both analysis using *p* < 0.001 and FDR correction.

We observed a negative correlation between GM volume and age in the left basal ganglia, olfactory cortex, hippocampus, lingual gyrus, and bilaterally in the cingulate; between GM volume and disease duration in the cerebellar lobules crus I, crus II, VII, VIIb, and VIII, in the left precuneus and middle frontal gyrus; between GM volume and BoNT in the left inferior temporal gyrus and between GM volume and MFS in the right inferior temporal gyrus, postcentral, and left rolandic operculum. There was a positive correlation between GM volume and disease duration in the cerebellar lobule VIII; between GM volume and BoNT in the left IFG, fusiform gyrus, superior parietal gyrus, postcentral gyrus, and cerebellar lobule IV/V and between GM volume and MFS in the left olfactory cortex and right precentral gyrus (Table 5).

**Table 5:** **Clinical correlation using *p* < 0.001 and *k* > 100 voxels**.

Clinical correlation *p* < 0.001				
MNI peak co-ordinates		
*x*	*y*	*z*	Voxels	Areas (automated anatomical labeling)
**Positive correlation: age and gm**				
No significant		
**Negative correlation: age and gm**				
−18	6	−14	175	Left olfactory cortex
			122	Left caudate nucleus
			101	Left lenticular nucleus, putamen
2	30	−2	879	Left anterior cingulate
			849	Right anterior cingulate
−20	−38	−1	182	Left hippocampus
8	−58	1	100	Right lingual gyrus
**Positive correlation: disease duration and gm**				
−20	−53	−40	400	Left cerebellar lobule VIII
14	−64	−37	782	Right cerebellar lobule VIII
**negative correlation: disease duration and gm**				
−20	−68	−35	3240	Right cerebellar lobule crus II
			1699	Left cerebellar lobule VIII
			1699	Right cerebellar lobule crus I
			1525	Left cerebellar lobule crus I
			966	Right cerebellar lobule VII
			836	Right cerebellar lobule VIII
			607	Cerebellar vermic lobule VII
			535	Cerebellar vermic lobule VIII
			385	Left cerebellar lobule VIIb
			305	Left cerebellar lobule crus II
			221	Cerebellar vermic lobule VI
			200	Left cerebellar lobule VI
−6	−49	57	104	Left precuneus
−24	20	48	112	Left middle frontal gyrus
**Positive correlation: bont and gm**				
−32	−23	−27	141	Left fusiform gyrus
−22	−40	−22	126	Left cerebellar lobule IV/V
−46	20	48	304	Left inferior frontal gyrus, triangular part
−22	−56	43	201	Left superior parietal gyrus
−38	−314	50	481	Left postcentral gyrus
**Negative correlation: bont and gm**				
−46	−17	−22	140	Left inferior temporal gyrus
**Positive correlation: mfs and gm**				
0	−83	23	198	Left olfactory cortex
46	2	59	260	Right precentral gyrus
**Negative correlation: mfs and gm**				
48	−28	−23	280	Right inferior temporal gyrus
−46	−12	13	104	Left rolandic operculum
54	−19	36	193	Right postcentral gyrus

## Discussion

Our results reveal decreased GM mostly in the premotor cortex (PMC), somatosensory integration areas, anterior cingulated/paracingulate, and cerebellum. Our findings were slightly more prominent in the left hemisphere, and we failed to detect any abnormality in the basal ganglia. These results share some interesting similarities with the most recent reports, while very discrepant ones from older studies. From the eight published studies in CCD using VBM (9–16), the earlier ones show varying results regarding increase and decrease in GM in the basal ganglia, while the later ones have not shown any abnormality in the basal ganglia (14, 15). Areas with consistent GM that decreases between the present study and prior ones are the SMA (9, 11, 16), PMC (9, 16), precentral gyrus (11, 15, 16), inferior parietal gyrus (13), superior temporal gyrus (12), visual cortex (9), precuneus, and the middle temporal gyrus (11). A recent abstract described decreased GM density in the rolandic operculum and insula in patients with CD and decreased GM in the precentral gyrus, cerebellum, operculum, cingulum, lingual gyri, and hippocampus in patients with BSP (18). There was also no atrophy in the basal ganglia or areas of GM increase.

Several possible explanations may account for the abovementioned discrepancies. First, there were multiple updates in the software used to perform the VBM analysis as well as new methodological protocols for VBM processing. Second, the widespread use of 3 T or stronger MRI scanners renders a more detailed and trustworthy image than the 1.5 T analysis that was used by seven of the eight previous studies (9–14, 16). Their difference in the VBM process, however, has never been definitely substantiated by later publications (19). Third, we used a larger control group to increase power. Prior studies mostly included one type of focal dystonia, either CD (9, 11, 12, 16) or BSP (12–15). Our study population was more heterogeneous and included individuals with broader clinical manifestations, including some never previously studied by imaging (such as oromandibular dystonia) and not only focal but also segmental dystonias. Our less restrictive inclusion criteria likely increase the external validity of our results, without compromising its internal one.

The precentral gyrus is involved in the execution of movements by activation of the motorneurons. The SMA and the premotor area are responsible for motor sequencing and planning (20). Information cascades downwards from the supramodal prefrontal cortex (PFC) to the primary motor cortex converting abstract goals in the PFC into motor plans in the premotor system. Thus, the PFC seems to play an important role in motor planning and learning (21). The PFC seems also to participate in changes or stops in voluntary movements by the inhibitory orbitofrontal impulses (22). The pars opercularis of the IFG is known for its role in speech production as well as speech programing and possibly the fluency and sequencing of the speech (23).

We found significant structural changes in the rolandic operculum, where the secondary somatosensory cortex resides and in the precuneus (mesial extent of Brodmann’s area 7 – part of somatosensory association cortex). Patients with CCD have abnormalities in the structures subserving sensation demonstrated by several studies using fMRI, PET, and electrophysiological techniques (24–26), while subjects with CD and BSP demonstrated a significant increase of spatial discrimination thresholds in both hands, suggesting abnormal sensory processing (27). Conversely, sensory tricks are considered a clinical manifestation of sensory involvement. The insular cortex is anatomically an elongation of the secondary somatosensory cortex and it is functionally connected to it as well as to the adjacent primary somatosensory cortex (28). The insula plays a central role in pain perception (29). Despite this sensory modality being rarely addressed in imaging studies in dystonia, it is a frequent symptom in dystonia, especially in CD. The abnormal responsiveness of the sensory-motor cortex seems to be driven by an unbalanced mechanism between long-term potentiation and long-term depression, leading to a maladaptative neuroplasticity activity, which is difficult to depotentiate in the motor cortex. The consolidation of abnormal motor engrams due to this mechanism may culminate in the overt muscular contraction observed in dystonic patients (30).

The sensorimotor network (SMN) comprised regions in the PFC, PMC, primary sensorimotor cortex (SM1), and the secondary somatosensory cortex, including the superior parietal lobule. We did not find any changes in the SM1. However, our study revealed small clusters of GM decrease in this area (data not shown) that might reach significance in a larger sample. This network seems to correspond mostly with the action–execution and perceptual–somesthesis paradigms (31, 32). The primary visual network (PVN) includes the PFC, PMC, SM1, superior parietal lobule, paracingulate gyrus, visual cortex, and middle temporal gyrus. Our study detected extensive GM decrease in both sensorimotor and PVNs. In CD, a task-free fMRI study showed decreased connectivity within these networks (33), and VBM, magnetization transfer imaging, and fractional anisotropy analyses presented changes in the visual areas (9, 11).

The PVN plays an important role in visual perception and processing of spatial information. Together with the frontoparietal network, they are responsible for building a representation of the space, in which spatial body knowledge is integrated from visual, proprioceptive, and somatosensory information, all crucial for accurate movement (34). The cingulate and precuneus are critical structures for the proper performance of the visuospatial system. Recent functional imaging findings suggest a central role for the precuneus in a wide spectrum of highly integrated tasks including spatial relations for body movement control (motor imagery) (35). Subjects with idiopathic dystonia have significant visuospatial function deficits (36) and the decreased connectivity within this system may manifest as abnormal head posture or movements (33).

We observed GM decreases in structures that are functionally associated with emotional processing and psychiatric disorders. Sixty percent of subjects with dystonia show psychiatric symptoms, and 42–71% of them developed those prior to the motor ones (37–39). As we did not perform a detailed neuropsychiatric evaluation, we cannot comment on this possible correlation.

The lack of substantial basal ganglia involvement in dystonia is not necessarily surprising. VBM is not the most accurate neuroimaging technique to verify morphometric changes in the deep cerebral GM. Earlier VBM studies reported contradictory results, with some showing GM reduction (12, 16) and others GM increase (12, 13). It is possible that, albeit a functional involvement of the basal ganglia is necessary to the development and the maintenance of the dystonic movement, an anatomical one is not imperative. Secondary dystonia may develop in subjects without basal ganglia damage (40, 41). Moreover, studies after basal ganglia lesions observed delays in symptom onset from weeks to years, indicating that the loss of function was not directly a consequence of the lesion itself, but rather from the process of neuroplasticity (42).

The regions of cerebellar involvement are a little different from our recent evaluation of the cerebellum using the SUIT tool for VBM in which we detected GM increase in the left cerebellar hemispheres I–IV and GM decrease bilaterally in the lobule VI, crus I, and right lobule VIIIb (7). In the first study, we used a specific tool for the cerebellum, since it provides a better overlap of the cerebellar lobules, preserves the anatomical detail, and does not allow the supratentorial structures to bias the results.

**Figure 2:**
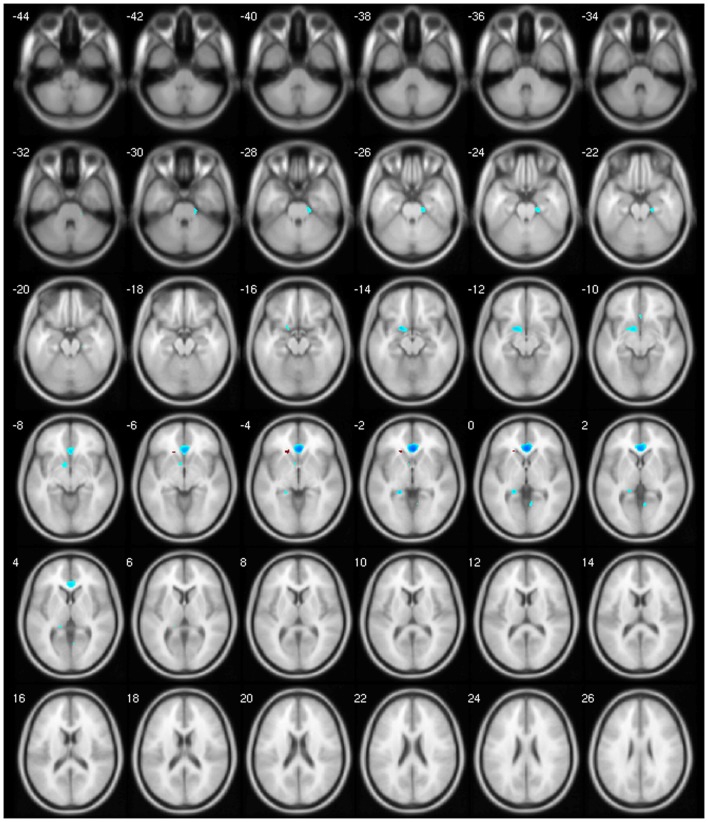
**Negative correlation between age and GM volume**.

**Figure 3:**
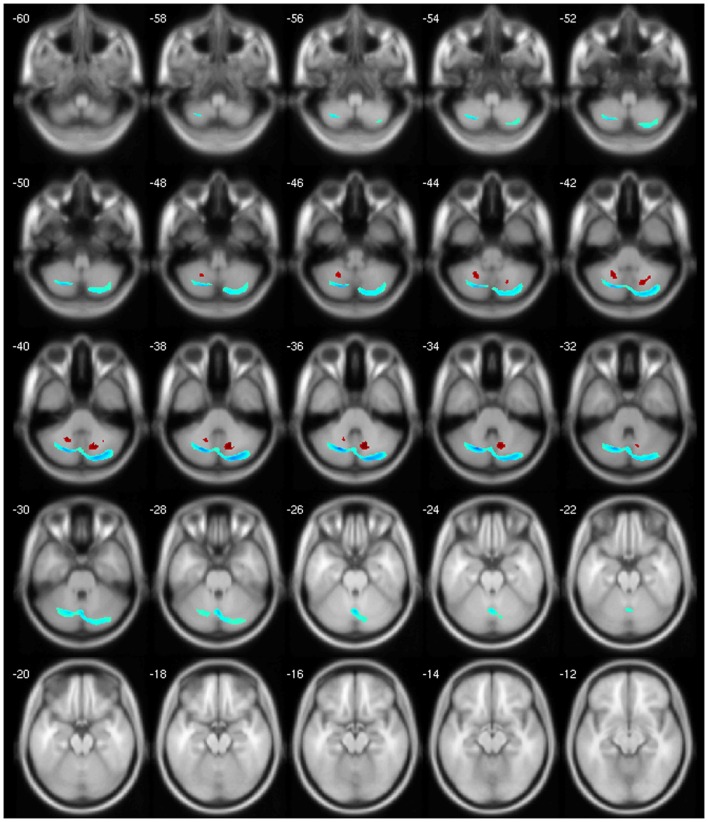
**Negative (blue) and positive (red) correlation between disease duration and GM volume**.

**Figure 4:**
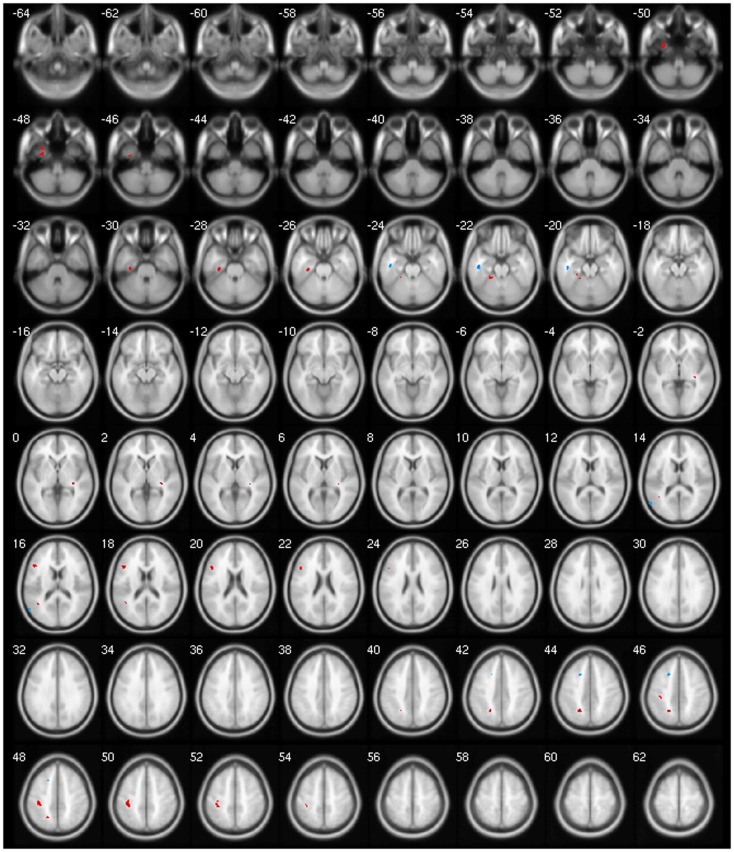
**Negative (blue) and positive (red) correlation between BoNT duration and GM volume**.

**Figure 5:**
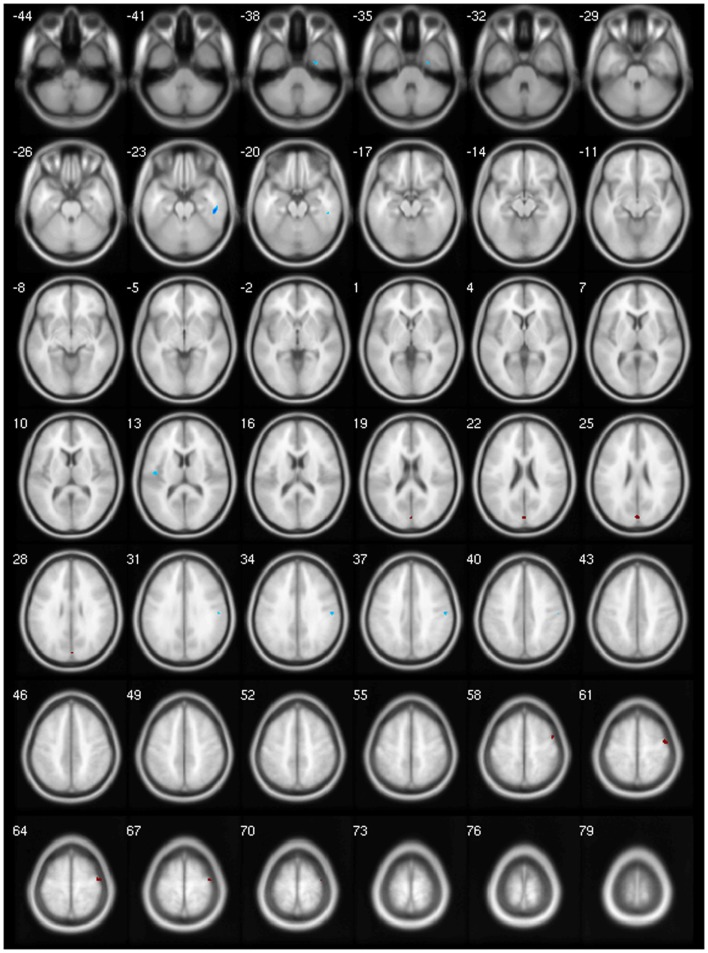
**Negative (blue) and positive (red) correlation between MSF score and GM volume**.

Finally, our clinical correlation showed some significant associations. The correlation between GM volume and age was negative for several areas, which we expected, since several VBM studies have identified age-related neocortical and subcortical volume reductions during adult life (43) (Figure 2). GM volume and disease duration were negatively correlated with the cerebellar lobules crus I, crus II, VII, VIIb, and VIII, in the left precuneus and middle frontal gyrus and positively correlated with cerebellar lobule VIII (Figure 3).The negative correlation between GM volume and the posterior cerebellar lobules reinforces the role of the cerebellum in dystonia as previously mentioned and published (7). Moreover, the correlation found suggests that the maintenance of dystonic movements has a negative impact in the cerebellar function leading to GM atrophy, which might be secondary to the circuit dysfunction. However, most of the previous studies failed to detect any clinical correlation. There is one longitudinal study only, which found GM decrease in the sensorimotor area in the 5-year follow-up (16). Therefore, further clinical correlation and longitudinal studies, as well as connectivity studies should be performed in order to clarify the trend of decreasing GM volume. GM volume and duration of BoNT were negatively correlated in the left inferior temporal gyrus but positively in the left fusiform, superior parietal, post central gyri, IFG, and cerebellar lobule IV/V (Figure 4). Delnooz et al. performed a longitudinal study comparing patients pre- and post-BoNT and showed an increased GM in patients post-treatment in the right dorsal PMC, inferring that BoNT is able to induce GM changes (44). Higher scores at MFS also showed both, a positive correlation in the left olfactory cortex and right precentral gyrus and a negative correlation in right inferior temporal gyrus, postcentral gyus, and left rolandic operculum (Figure 5). It is very interesting to observe that subjects with more severe disease have more atrophy at the postcentral gyrus.

The major limitation in our study is the heterogeneous collection of dystonias. However, there is a significant clinical overlap between those forms of dystonia, which is observable in subjects with progression of the dystonia to continuous segments. Some studies have also studied CCD as a group (2–7). Our aim was not to evaluate the differences between those, but rather to elucidate common mechanisms in the pathophysiology of CCD, which could even be hypothesized in other presentations, such as task-specific and focal hand dystonia (45, 46). Another limitation is the lack of proper clinical characterization of the side of the symptoms and documentation of hand dominance in our subjects. Previous studies showed asymmetrical interhemispheric activity along the direct pathway within the basal ganglia (47). A technical limitation is that the SUIT tool used in our previous analysis cannot be combined with the whole-brain template, which led us to perform two separate analyses. Unfortunately, our results can only be considered exploratory, since, a more strict correction (FDR and FWE) did not reveal any significant result.

## Conclusion

We detected diffuse involvement in CCD. The GM changes in the premotor, motor, and somatosensory cortex corroborate the disorganization of the SMN. The structural involvement of the frontoparietal network, the visual areas, the precuneus, and the cingulated are potentially involved in the visuospatial impairment presented in patients with dystonia and have been recently described by several authors. Finally, the insula, the limbic system, and the cerebellum elucidate possible contributory mechanisms in the pathophysiology of the dystonia. Nonetheless, it is unclear if the GM findings observed are primary or secondary, and further longitudinal studies may be better suited to answer this question.

## Author Contributions

(1) Research project: A. conception: Anelyssa D’Abreu; Camila C. Piccinin; Maria C. A. Santos; Iscia Lopes-Cendes; Fernando Cendes. B. organization: Anelyssa D’Abreu; Camila C. Piccinin; Maria C. A. Santos. C. execution: Camila C. Piccinin; Maria C. A. Santos; Luiza G. Piovesana; Lidiane S. Campos; Rachel P. Guimarães; Brunno M. De Campos; Thiago J. R. Rezende; Fabio R. Torres; Marcondes C. França Jr.; Augusto C. Amato-Filho/Anelyssa D’Abreu. (2) Statistical analysis: A. design: Anelyssa D’Abreu; Brunno M. De Campos; Rachel P. Guimarães; Luiza G. Piovesana; Fabio R. Torres; Thiago J. R. Rezende; Iscia Lopes-Cendes; Fernando Cendes. B. execution: Camila C. Piccinin; Maria C. A. Santos; Brunno M. De Campos; Luiza G. Piovesana; Rachel P. Guimarães. C. review and critique: Anelyssa D’Abreu; Marcondes C. França Jr.;Thiago J. R. Rezende; Iscia Lopes-Cendes; Fernando Cendes. (3) Manuscript: A. writing of the first draft: Camila C. Piccinin; Anelyssa D’ Abreu. B. review and critique: Anelyssa D’Abreu; Iscia Lopes-Cendes; Fernando Cendes; Luiza G. Piovesana; Lidiane S. Campos; Rachel P. Guimarães; Fabio R. Torres; Marcondes C. França Jr.; Augusto C. Amato-Filho.

## Conflict of Interest Statement

None of the authors have conflicts of interest directly related to the development of the manuscript. Full financial disclosures can be found in the Acknowledgments section.
